# Emergence, self–organization and morphogenesis in biological structures

**Published:** 2011-02-25

**Authors:** R Dobrescu, VL Purcarea

**Affiliations:** * Faculty of Automatic Control and Computers, Polytechnic University of BucharestRomania; ** ‘Carol Davila’ University of Medicine and Pharmacy, BucharestRomania

**Keywords:** emergence, self–organization, morphogenesis, pattern formation, reaction–diffusion systems, fractal analysis

## Abstract

The paper discusses the connection between emergence, pattern formation and nonlinear dynamics, focusing on the similarity between discrete patterns and fractal structures, and then describes different solutions to model reaction–diffusion systems as representative processes in morphogenesis. A specific example is the diffusion limited aggregation growth process, illustrated by the simulation of the evolution of a bacterial colony that shows the roles of instability and sensitivity in non–equilibrium pattern formation. Based on this particular case, it is shown how self–organization could be achieved from non–organized agglomeration of separate entities, in a region of space. We conclude with some brief remarks about universality, predictability and long–term prospects for this field of research.

## Introduction

Our approach deals essentially with pattern formation in biological systems far from equilibrium state, trying to underline a connection between the general principles of morphogenesis, the dynamics of the reaction–diffusion systems and the fractal analysis as a tool for modeling such processes. 

What makes the present stage of biological science so extraordinary is that molecular biology is driving us to the innermost reaches of the cell's ultimate mechanisms, complexity, and capacity to evolve. At the very same time, work in Mathematics, Physics, Chemistry, and Biology is revealing how far–reaching the powers of self–organization can be. These advances hold implications for the origin of life itself and for the origins of order in the ontogeny of each organism. The order inherent in the busy complexity within the cell may be largely self–organized and spontaneous rather than the consequence of natural selection alone.

The variety of natural patterns makes it difficult to analyze and compare them in a systematic manner. We address this problem by focusing on the computational aspects of pattern formation processes. They are characterized in terms of the number of morphogenetic agents, the computing capability of each agent, and the forms of information transfer between the agents and their environment. This computational analysis can be applied to a wide range of patterns. It highlights the fundamental, algorithmic similarities between processes that may be implemented by using different physical or physiological mechanisms in nature. It also confirms earlier observations that fundamentally different processes can create similar or identical patterns. The tradeoffs between computational characteristics of these processes lend themselves to a formal analysis, which could lead to the formulation of a ‘computational theory of morphogenesis’ based on the theory of algorithms.

## A conceptual approach for emergence

Emergence is a fundamental property of complex systems and can be thought of as a new property or behavior, which appears due to non–linear interactions within the system; emergence may be considered the ‘product’ or by–product of the system. As our world becomes increasingly more interconnected, understanding how emergence arises and how to design for it and manage specific types of emergence is ever more important. To date, the concept of emergence has been mainly used as an explanatory framework [10], to inform the logic of action research [14] or as a means of exploring the range of emergent potential of simulation of real complex systems [1].

The ability of current models to fully portray emergence in all its possibilities has been questioned,   but current conceptual models of emergence (for example [6]) are too simplistic or general to be useful when examining the range of types of emergent phenomena observed in real physical, biological and social systems. Improved understanding and modeling of emergence is required. Through its nature, emergence is problematic to the model. It is the product of interconnections and the interaction makes it dynamic and unpredictable; entities, interactions, their environment and time are key contributors to emergence, even though there is no simple relationship between them. For example, self–organization, while linked with the appearance of hierarchical structures and system wide properties does not account for the emergence of true novelty or semantics and meaning. Are there underlying generalizations that can be drawn from the occurrences of different types of emergence, which can be applied to real systems? This is after all one of the underlying principles of Complexity Science. Another poorly understood issue is how emergent properties or dynamics appear to influence the behavior of the constituent entities of the system. A new model approach was adopted because it allowed a useful representation of salient features pertaining to the issues under investigation. Such a model does not necessarily have the explanatory power of formal models; it rather provides a working strategy, a scheme containing general, major concepts and their interrelations. 

In order to underline the connection between emergence and self–organization, one can discuss the features of the four meta–classes–first introduced in McDonald and Weir (2005) [15]. Features were considered potential meta–classes if (i) they were the product of non–linear interactions (ii) they were domain independent (iii) they were a core building block for system interactions and (iv) they open up new system potential by their existence. Self–organization, where detailed organizational structure and new system interactions emerge as a result of the behavior rules of the system entities, is a well documented feature of real complex systems. The term self–organization is varyingly used to describe the dynamics of complex systems, emergence or the specific organizational changes brought about through the autonomous entity behavior. The term self–organization is defined as the structural change in a complex system that arises from nonlinear, possibly noisy interaction. Because this structural change is a collection of parts with ordered asymmetric relationships, we class it as hierarchy. The concept of hierarchy again is well documented, frequently acting to constrain the degrees of freedom of a complex system.

As Kauffman observes, some emergence fundamentally changes the complex system in which it appears [12]. Therefore, we define novelty as the emergence of a sustainable new entity with distinctly different interaction patterns. For Pattee, the role of memory is crucial in biological systems–‘evolution depends, at least to some degree, on control of dynamics by rate–independent memory structures’ [17]. These memories must first appear before the complex systems and may capitalize on them. Therefore, we define the memory meta–class as frozen structure or processes that arise through non–linear interactions. Biological life relies on synergistic coupling, which happens in nature through an entity's ability to sample its environment and make use of synergistic opportunities–it uses other entities or processes within the system to do work. This is an example of what we call the emergence of functionality, in which a new process that carries out ‘work’ used by another entity emerges. Before entities can make use of other entities or processes in this synergistic way, they must be able to detect their existence. When a new ability for environment sampling arises through interaction, we define that the emergence of measurement. Related to the emergence of memory it is the issue of its accessibility to the various entities of the complex system. When the non–linear interactions cause this memory or processes within the system to be restricted to certain parts, we describe it as localization. If the localized memory or processes are used differently within the system, then the context is said to have emerged. The ability of the entities is to recognize patterns that trigger specific behavior, although the original causal pattern may be lost. That significantly adds to the creativity of complex systems. We call this symbolism.

Other concepts such as autocatalysis, reproduction, evolution, dissipative systems and autopoiesis, are not detailed because these are specialized forms of processes that emerge and therefore are included under function / process.

## Paradigms of pattern formation

### Definitions and classifications

First, let us accept a discussion only on discrete patterns, which are structures, based on repetitive occurrences of predefined figures, called motifs. A pattern is assumed invariant with respect to some isometries of the plane, called its symmetries. The act of setting all the symmetries forms a group under composition; in particular, fractal structures can be assimilated as discrete patterns. 

Fractal patterns abound in the natural world and include, to name but a few examples for which mathematical models have been proposed, branching patterns of plants and rivers, venation patterns in leaves, arrangements of cells in organs of plants and animals, pigmentation patterns in sea shells and buttery wings or animal coat patterns. 

**Figure 1:**
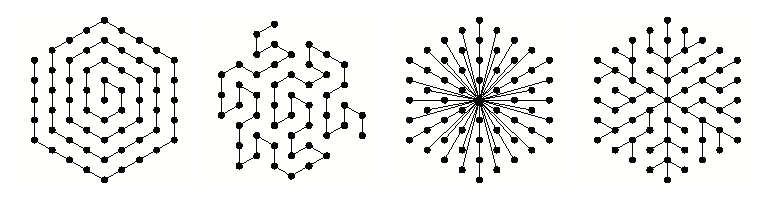
Typical patterns proposed by Stevens

Stevens distinguished four prototypical classes of patterns (see Figure 1: a) spirals, b) meanders, c) explosions, d) branching patterns) by considering different methods of connecting a set of regularly arranged points into a graph without cycles [20].

Patterns in each class were characterized by geometric attributes: uniformity, space filling, overall length of lines, and directness of lines. This characterization proved useful when analyzing natural patterns from the viewpoint of their optimality. Unfortunately, the optimality of the result does not offer a direct insight into the mechanisms that govern pattern formation. A classification that addressed this limitation was suggested by Bell, who distinguished the following three categories of branching patterns [2]:

- Blind patterns, in which branch initiation is controlled solely by the imposed program rules, that is without the ‘organism’ and its environment.
- Sighted patterns, in which the initiation of a new branch is influenced by factors detected by it in the immediate neighborhood, such as proximity of other organisms, or parts of the same organism.
- Self–regulatory patterns, in which the developing simulation itself controls branch initiation, using communication via components of the existing framework, whether or not affected by environmental factors.

Focusing on the fundamental, algorithmic properties of pattern formation, the above classification makes it possible to recognize and analyze similarities between apparently different realizations of similar patterns, and does not presuppose any computational framework for model construction. However, one of these models seems the most suitable to simulate pattern formation. It is the model of a reaction–diffusion systems, which is in the same time the most appropriate to describe the construction of a fractal structure [18]. 

### Bifurcations in pattern formation

After instability has produced a growing disturbance in a spatially uniform system, the next crucial step in the pattern–forming process must be some intrinsically nonlinear mechanism by which the system moves toward a new state. That state may resemble the unstable deformation of the original state. The system evolves in entirely new directions as determined by nonlinear dynamics. We now understand that it is here, in the nonlinear phase of the process, that the greatest scientific challenges arise. The inherent difficulty of the pattern–selection problem is a direct consequence of the underlying (linear or nonlinear) instabilities of the systems in which these phenomena occur. A system that is linearly unstable is one for which some response function diverges. This means that pattern–forming behavior is likely to be extremely sensitive to small perturbations or small changes in system parameters. Therefore, some important questions are: Which perturbations and parameters are the sensitively controlling ones? What are the mechanisms by which those small effects govern the dynamics of pattern formation? What are the interrelations between physics at different length scales in pattern–forming systems? 

Let us now present a possible strategy to answer these questions. In dynamical systems theory, the stable steady solutions of the equations of motion are known as ‘stable fixed points’ or ‘attractors’ and the set of points in the phase space from which trajectories flow to a given fixed point is its ‘basin of attraction.’ As the control parameters are varied, the system typically passes through ‘bifurcations’ in which a fixed point loses its stability and, at the same time, one or more new stable attractors appear. An especially simple example is the ‘pitchfork’ bifurcation at which a stable fixed point representing a steady fluid flow, for example, gives rise to two symmetry–related fixed points describing cellular flows with opposite polarity. 

The theory of bifurcations in dynamical systems helps us understand why it is sometimes reasonable to describe a system with infinitely many degrees of freedom using only a finite (or even relatively small) number of dynamical variables. It is for this reason that we may need only a low–dimensional space of dynamical variables to describe some pattern–formation problems near their thresholds of instability–a remarkable physical result.

## Modeling reaction–diffusion systems

### The starting point

Qualitative studies of reaction diffusion systems of equations have probably begun in 1952. The reason is that two paradoxes of diffusion were demonstrated by nonlinear differential equations in the same year–1952. The first was shown by a mathematician, A. Turing, who is well known as a great pioneer in the field of computer science. He suggested, by using a simple reaction–diffusion (RD) system, a paradox that diffusion enhances spatial in–homogeneity, although we know, as common sense, that diffusion does enhance homogeneity in space [21]. He also claimed that such ‘diffusion–induced instability’ gave the possibility to play a role in the mechanism of cell differentiation and morphogenesis arising in the field of developmental biology. Two neurophysiologists, A. L. Hodgkin and A. F. Huxley, who investigated the mechanism of impulses propagating along nerve fiber [8], suggested the second one. One of the physiological problems was to clarify the reason why nerve impulse constantly propagates with fixed shape. In the same year Turing' paradox was stated, they proposed a model of nonlinear partial differential equations, which is given by the coupling of a single RD equation with three ODEs in order to describe the propagation of impulses along the fiber. [16] Unfortunately, it was so hard to analyze their model at that time, because it contains high nonlinearity. However, their model could be numerically solved by using computer calculation. It is surprising that this model generates a traveling pulse wave with constant shape as well as velocity, in spite that it is described by diffusion equations. This indicates another paradoxical evidence of diffusion, that is, suitable RD systems possibly generate a localized wave.

**Figure 2:**
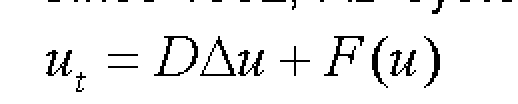
RD systems

Since 1952, RD systems have had the form:(Figure 2)

**Figure 3:**
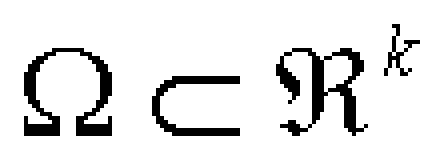
Domain

They have been intensively investigated in the fields of not only applied science such as Biology, Chemistry, Physics but also in Mathematics. In a general case, equ. (1) models the diffusion through a domain (Figure 3)

of m interacting species or chemicals, where the i–th component u_i_ of u = (u_1_,…, u_m_) represents the density or concentration of the i–th reactants and D = (d_1_, …, d_m_) is the matrix of the diffusion constants d_i_ > 0 [9]. One hard mathematical task for the practical use of these models is to find the appropriate vector supply terms F in such a way that the pattern formation process, governed by the corresponding reaction–diffusion system, coincides with the phenomenon observed in the laboratory experiments or in nature. 

Because the pattern formation process is the main subject in question, we can ask if the complexity of patterns modeled by the reaction–diffusion systems can be arbitrary. A pattern is the eventual result of a time evolution of a biological, chemical, or physical process and thus has the following two main features: a) Long–time effect and b) Great randomness of the initial conditions. Based on this observation, we saw that a pattern is a kind of attractor. By an attractor for a reaction–diffusion system we mean the mathematical object, which attracts an open set of initial data in such a way, that the trajectories starting from this initial data set eventually, end up on the attractor in question (this is just the long–time effect of an attractor). The openness of the set of initial data guarantees the required great randomness of initial data, which lead to the same pattern (attractor). Moreover, this openness corresponds to the practical need (i.e., for the computer simulation) that there is a positive probability that computed trajectories will tend to the attractor. Using the concept of attractors, well known in fractal analysis, the above universality problem can be mathematically more exactly reformulated as it follows Can the complexity of attractors for the reaction–diffusion systems be arbitrary?

Further, we will give a method of constructing the vector supply term F for the purpose that the corresponding reaction–diffusion system has an attractor, whose complexity can be arbitrary in some sense. 

### Model construction

**Figure 4:**
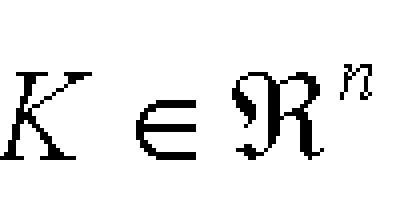
K included in R^n^

Let and n be included in N and Figure 4 be a given connected compact subset of arbitrary complexity. Then there is a vector supply term FK of the form:

**Figure 5:**
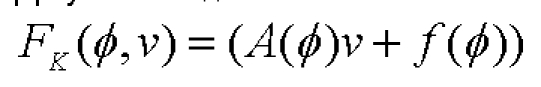
vector supply term FK

for Figure 6 and Figure 7 where A is a smooth function on R and f a smooth map on R^n^, such that the corresponding reaction–diffusion system

**Figure 6:**
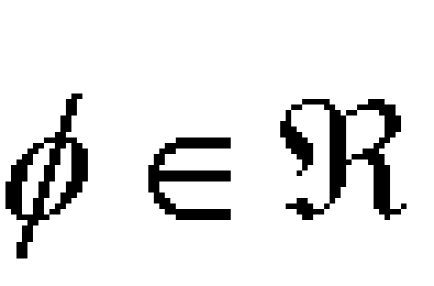
form 1

**Figure 7:**
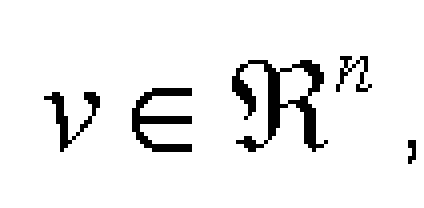
form 2

**Figure 8:**
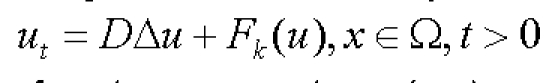
reaction diffusion system

 of n+1 components u=(०,v), accompanying with the zero flux boundary condition, generates a dynamical system in the state space C(ष)^1+n^ with the following properties:

- For each initial value u_0_  C(ष)^1+n^ the reaction–diffusion system has a global unique solution u, u(0) = u_0_, such that u is continuous in षx[0,蜴), and u_t_, खu as well as all partial derivatives are continuous in षx(0,蜴).
- Each solution u of the reaction–diffusion system starting from an initial value u_0_=(०_0_,v_0_)  C(ष)^1+n^ such that either ०_0_>0 or ०_0_<0 is asymptotically stable and converges to the set Figure 9

**Figure 9:**
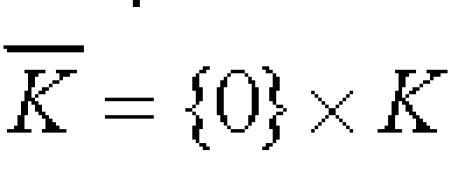
set2

in the sense that ३(u,C)=Figure 10.

**Figure 10:**
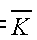
form 3

**Figure 11:**
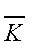
set

As result, the connected compact set Figure 11 

is an attractor for the reaction–diffusion system settled in the state space C(ष)^1+n^.

**Figure 12:**
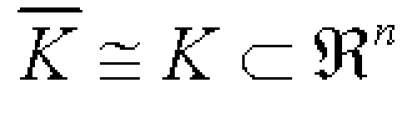
pattern

Here we chose the zero flux boundary condition and the positive initial condition, since they are probably the most interesting boundary and initial conditions in the biological or chemical situation. Namely, the zero flux boundary condition reflects the self–organization mechanism of pattern while the positive initial condition restricts the pattern formation process to such a beginning circumstance that each of the reactants has a positive distribution all over the reaction domain. Thus, our statements imply that any pattern (here K) which is isomorphic to a connected compact subset (here K) of the Euclidean space R^n^ can be seen as the final result of the pattern formation process governed by some appropriate reaction–diffusion system of n + 1 components. Moreover, it implies the following assertion: The make–up of a pattern Figure 12 

with arbitrary complexity (i.e., a fractal pattern [4] can be realized by a reaction-diffusion system of the form (3) once the vector supply term F_K_ has been previously properly constructed.

### A model of the dendritic pattern formation process

Although the above construction is the product of theoretic thoughts, we were also interested in whether it is possible to derive such reaction-diffusion systems from any sequence of reasonable biochemical or physical situations, where one can consider that the first component ० of the vector u=(०,v) works as the activator and the rest components v as the inhibitor of the system). This mechanism simplicity throws light on the possibility of deriving reaction–diffusion systems of the form (3) from real world situations. One of these particular real world examples is the process of diffusion–limited aggregation (DLA) encountered in dendritic pattern formation [7].

The dendritic pattern formation process can be observed in different areas of non–equilibrium pattern formation: Metallurgy (dendritic solidification), Medicine (tumor growth), Biology (bacterial colony development) and so on. In the most common situations, dendritic growth is controlled by diffusion–either the diffusion of latent heat away from the growing solidification front or the diffusion of chemical constituents toward and away from that front. These diffusion effects very often lead to shape instabilities; small bumps grow out into fingers because, like lightning rods, they concentrate the diffusive fluxes ahead of them and therefore grow out more rapidly than a flat surface. Today's prevailing theory of free dendrites is generally known as the ‘solvability theory’ because it relates the determination of dendritic behavior to the question of whether or not there is a sensible solution for a certain diffusion–related equation that contains a singular perturbation. The term ‘singular’ means that the perturbation completely changes the mathematical nature of the problem whenever it appears, no matter how infinitesimally weak it might be. In the language of dynamical systems, the perturbation controls whether or not there is a stable fixed point. Similar situations occur in fluid dynamics, for example, in the ‘viscous fingering’ problem [13]. The theory has been checked in numerical studies that have probed its nontrivial mathematical aspects [11]. As a result, although we know that there must be other cases (competing thermal and chemical effects, for example), we now have reason to confide that we understand at least some of the basic principles seen in fluids and granular materials correctly. The degree to which we can develop quantitative, predictive models of these phenomena will determine the degree to which we can control them and perhaps develop entirely new technologies. As an example, in the next section of the paper we will discuss a simulation of the dynamics of a predictive model of pattern forming in a biologic system. 

**Figure 13:**
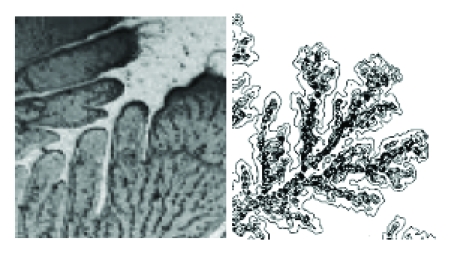
Structure of ordered aggregates within branches (a) microscopic view; b) simulated growth).

This simulation model tries to reflect the real behavior of cells observed microscopically.  Looking through the microscope at colonies of a certain T morphotype [3], one can see cells performing a random–walk–like movement in a fluid. We assume that this lubrication fluid is excreted by the cells and/or drawn by the cells from the agar culture medium. The cellular movement is confined to this fluid; isolated cells spotted on the agar surface do not move. A closer look at an individual branch (Figure 13) reveals a phenomenon of density variations within the branches. These 3–dimensional structures arise from accumulation of cells in layers. The aggregates can form spots and ridges which are either scattered randomly, ordered in rows, or organized in a leaf–veins–like structure. The aggregates are not frozen; the cells in them are motile and the aggregates are dynamically maintained. The picture shows variation in the height of the branches. The more bacteria are in a unit area, the more layers the bacteria are in, and the higher the area seems. Thus, the boundary of the fluid defines a local boundary for the branch. Whenever the cells are active, the boundary propagates slowly as a result of the cellular movement pushing the envelope forward and due to the production of additional wetting fluid. Electron microscope observations reveal that these bacteria have flagella for swimming. The observations also reveal that the cells are active at the outer parts of the colony, while closer to the center, the cells are stationary and some of them sporulate. It is known that certain bacteria respond to adverse growth conditions by entering a spore stage until more favorable growth conditions return. Such spores are metabolically inert and exhibit a marked resistance to the lethal effects of heat, drying, freezing, deleterious chemicals, and radiation. 

## Model implementation and simulation results

### Model design

We can gain much insight into instability mechanisms and nonlinear states from the continuous models of biological processes. Often, though, it is more convenient to compute with discrete ‘automata’ models, which in some sense are designed to be simulated. In fact, we will see that perhaps the most convenient approach for microbial systems seems to be a hybridization of continuum and atomistic methods. Let us start with systems exhibiting diffusive instabilities. Initially, the simplest discrete analogue was afforded by diffusion–limited–aggregation (DLA). Here, discrete walkers move diffusely in space and attach to a growing cluster. In the limit of taking one walker at a time (i.e. of extremely slow growth) and purely irreversible attachment at any nearest–neighbor site, one obtains the classic DLA fractal [21]. It is well worth emphasizing the beneficial aspects of having a connection between a discrete simulation and a related continuum model. It is usually difficult to do much beyond simulation for a discrete model; so, having a continuum analogue allows an analysis that helps guide the simulations and vice versa. Once the basics are understood, one can modify the simulation to encompass more details of the actual system and thereby obtain results that are more reliable. But, just doing the simulations or even the analysis is insufficient; one must understand the fundamental mechanisms at the core of an observed simulated structure as these can then be compared to the true underlying biological dynamics.

Aside from computational convenience, there are good reasons why the modeling of biological systems can make good use of discrete entities. First, the numbers match more closely. Perhaps more importantly, cells contain large numbers of internal degrees of freedom, which modulate their response to external signals from other cells. Hence, describing a population of cells with something as non-informative as a density field is usually insufficient. At the very least, one would have to introduce either new variables or even new coordinates. Tracking cells as individual objects makes it easy to do this; we just attach extra labels to the cell and postulate transition rules as to how these labels change in time. This flexibility is quite useful and hence some of the models to be discussed keep cells discrete. At the same time, though, continuum analysis is used to shed light on the simulations and forms an indispensable part of an integrated effort to understand microbiological pattern formation.

The Discrete Walkers (DW) model describes the growth of colonies of T morphotype. The model was inspired by the diffusion-transition scheme proposed by Cohen in his Ph.D. thesis [3]. This scheme is a hybridization of the ‘continuous’ and ‘discrete’ approaches used in the study of non–living systems. In the DW model, discrete walkers that obey dynamic rules represent the bacterial cells. The DW model also consists of at least one chemical field, namely nutrient concentration field, and additional element such as a free boundary of the colony. A walker in the DW model does not represent a single bacterium. Each of the walkers will usually be taken to represent about one hundred cells. Each of the walkers has a position r_i_ and a metabolic state H_i_. The lubrication fluid is not incorporated as such into the model, only its effects on the bacterial movement. The area occupied by the colony (wetted by the lubrication fluid) is defined by an on–lattice boundary representing the boundaries of the layer of lubrication fluid. To incorporate the swimming of the bacteria into the model, the walkers perform an off–lattice random walk within the area already occupied by the colony. At each time–step each of the active walkers attempts to move from its location a step of size d at a random angle ॒(॒ chosen from [0;2ॠ] with uniform distribution), to a new location r' given by: r'=r+dcos॒. Although d is used in this equation as if it has length units, its units are actually the square root of the units of a diffusion coefficient. These units compensate for fact that the number of steps of a walker per time unit is sensitive to the time–step of the model's simulation. If the units of d would have been length units, then the effective diffusion coefficient of the walkers in the bulk of the colony would have been sensitive to the time–step of the model's simulation. If the new location r' is outside the boundary, the walker does not perform that step, and a counter on the segment of the boundary which would have been crossed by the movement from r to r' is increased by one. When the segment counter reaches a pre–specified number of hits N_c_, the boundary propagates one lattice step and an additional lattice cell is added to the area occupied by the colony. N_c_ is measured in units of length to the power of–D (where D is the spatial dimension of the simulation: 2 or 3). The requirement of Nc hits represents the colony propagation through collective production of lubrication fluid and wetting of unoccupied areas. N_c_ is directly related to the food concentration, as more lubrication fluid has to be produced to push the boundary on a drier substrate.

**Figure 14:**
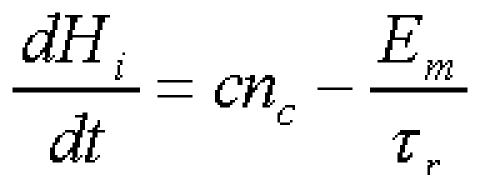
Dynamics of the energy

**Figure 15:**
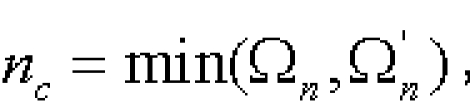
Nutrient consumption rate

We represent the metabolic state of the i–th walker by an ‘internal energy’ H_i_. The dynamics of this energy is given by Figure 14

where c is a conversion factor from nutrient to internal energy and E_m_ represents the total energy loss for all processes (excluding reproduction) over the minimal time of reproduction T_r_. The nutrient consumption rate n_c_ is Figure 15

where ष^n^ is the maximal rate of nutrient consumption of a walker, and ष'n is the rate of nutrient consumption as limited by the local availability of nutrient. The maximal rate of nutrient consumption of a walker equals the consumption rate per cell times the number of cells represented by a single walker. When sufficient nutrient is available, H_i_ increases until it reaches a threshold energy Ed and the walker divides into two. When the walker is ‘starved’ for a long interval of time, H_i_ drops to zero and the walker ‘freezes’. This ‘freezing’ represents the transition into pre–spore state. For simplicity, we have assumed in our experiments that the cellular density is suitable for sporulation, so that the limiting factor is the supply of nutrients.

### Experimental results

**Figure 16:**
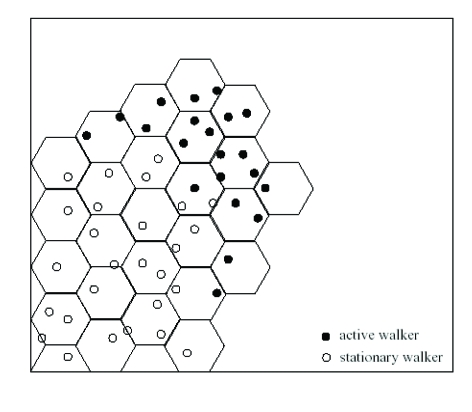
A branch in a simulation of the DW model

**Figure 17:**
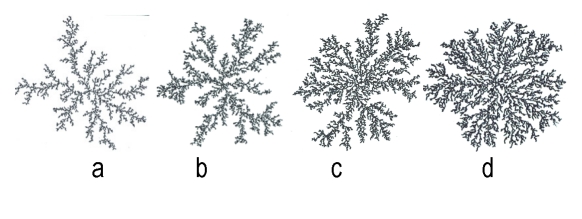
Simulated colonial patterns of the DW model

The diffusion equation is solved on a triangular lattice with a lattice constant खx, the same lattice on which the boundary is outlined. For numerical stability the walkers' step length,d蜰खt(where खt is the simulation's time–step), must be smaller than the lattice constant. All the simulations are stopped when the colony reaches a given radius. Results of numerical simulations of the model are shown in Figure 16 (microscopic view: the hexagons are those lattice cells that were occupied by walkers and became part of the colony; the reaction–diffusion equations are solved on the whole lattice, weather part of the colony or not) and Figure 17 (colonial patterns, with N_c_ = 20 and the conversion factor c is 6 (a), 8 (b), 10(c) and 30(d) from left to right respectively). As in real bacterial colonies, the simulated patterns are compact at high nutrient concentration levels and become fractal with decreasing nutrient level. For a given nutrient level, the patterns are more ramified as the food concentration increases.

**Figure 18:**
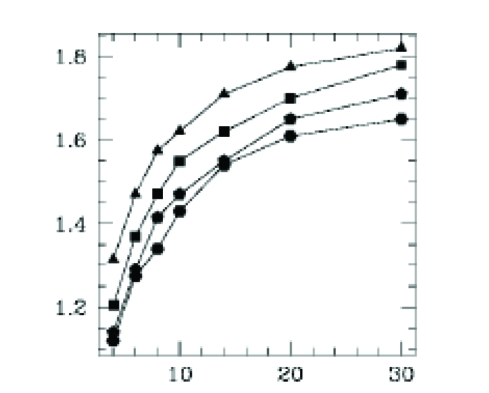
Fractal dimension as a function of initial food concentrations (triangle–N_c_=10;square–N_c_=20;2.rhombus –N_c_=30; round–N_c_=40)

Figure 18 shows the qualitative dependencies of the fractal dimension of the nutrient concentration levels. Clearly, the results are encouraging and do capture some features of the experimentally observed patterns. The branching patterns are a manifestation of the diffusion field instability. From this perspective, it is quite reasonable that the effect of the instability is enhanced as the food concentration is raised and the motion of the bacteria is suppressed. This is analogous to lowering the diffusion coefficient of a bacterial density field (in a continuum description), which leads further to the diffusively unstable region of the parameter space.

One can explain why the velocity of the fine radial branching patterns is lower than that of the fractal growth observed at higher levels. We assume that for the colonial adaptive self–organization, the T morphotype employs three kinds of chemotactic responses. One is the food chemotaxis, which is expected to be dominant for a range of nutrient levels (the corresponding levels of nutrient are determined by the constant K). The two other kinds are self–induced chemotaxis or signaling chemotaxis, i.e. chemotaxis towards or away from chemical produced by the bacterial cells themselves. For efficient self-organization it is useful to have two chemotactic responses operating on different length scales, one regulating the dynamics within the branches (short length scale) and the other regulating the organization of the branches (long length scale). The length scale is determined by the diffusion constant of the chemical agent and the rate of its spontaneous decomposition. If there is also decomposition of the chemical by the cells, it gives them additional control of the length scale. 

## Self–organization and morphogenesis in biological systems

The principles of construction in complex integrated systems of elements that allow the systems to adapt their behavior in a complex environment can be summarized in two themes: first, the emergence of profound spontaneous order; second, a bold hypothesis that the target of selection is a characteristic type of adaptive system poised between order and chaos. The unexpected spontaneous order is this: vast interlinked networks of elements behave in three broad regimes–ordered, chaotic, and complex regime on the frontier between order and chaos. The spontaneous order of the ordered regime foretells much of the order seen in aspects of developmental biology. Ordered systems, particularly those near the edge of chaos, have the needed properties. Moreover, we see that the same construction requirements find echoes at higher levels, such as whole ecosystems. Here the problem is to understand how such systems are coupled so that members coevolve successfully and how selection itself may achieve such coupling. Again, such ecosystems can behave in three broad regimes –ordered, complex and chaotic. Again, remarkably, coevolving systems may optimize their capacity to coevolve by mutually attaining the edge of chaos.

Any major example of powerful self–ordering shows that in each case, the spontaneous order appears so impressive that it would be shortsighted to ignore the possibility that much of the order we see in the biological world reflects inherent order.

Now, let us examine the ‘genetic program’ which controls cell differentiation during development of the adult from the fertilized ovum, and the machinery that yields ordered morphologies. The main intent is to suggest that many highly ordered features of ontogeny are not the hard–won achievements of selection, but largely the expected self–organized behaviors of these complex genetic regulatory systems.

The problem of cell differentiation is one of the two most basic issues in developmental biology. Different cell types–nerve, muscle, liver parenchyma–arise and differentiate from earlier cell types during development and, ultimately, in a human, form several hundred–cell types. Each cell in the human's body essentially contains the same genetic instructions as all the other cells. Those instructions include the structural genes coding for about 100 000 different proteins. Cell types differ because different subsets of genes are ‘active’ in the different cell types. The activation and repression of genes is itself controlled by an elaborate regulatory network in which the products of some genes switch other genes on or off. More generally, the expression of gene activity is controlled at a variety of levels, ranging from the gene itself to the ultimate protein product. It is this web of regulatory circuitry that orchestrates the genetic system into a coherent order.

That circuitry may comprise thousands of molecularly distinct interconnections. In evolution, the very circuitry is persistently ‘scrambled’ by various kinds of mutations, as the ‘logic’ of the resulting developmental program is. It is important to note that the main properties such as the existence of distinct cell types, the homeostatic stability of cell types, the number of cell types in an organism, the similarity in gene expression patterns in different cell types, the fact that development from the fertilized egg is organized around branching pathways of cell differentiation, and many other aspects of differentiation are all consequences of properties of self organization, so profoundly immanent in complex regulatory networks whose order selection cannot avoid. All aspects of differentiation appear to be properties of complex parallel–processing systems lying in the ordered regime [19]. These properties may therefore reflect quasi–universal features of organisms due not to selection alone, but also to the spontaneous order of the systems on which selection has been privileged to act.

Now, let us discuss the second fundamental problem in developmental biology: morphology. The actual morphologies of organisms must also be viewed as a collaboration between the self–ordered properties of physico–chemical systems together with the action of selection. Oil droplets are spherical in water because that is the lowest energy state. Thus the genome's capacity to generate a form must depend on very many physico–chemical processes constituting a panoply of developmental mechanisms beyond the sheer capacity of the genome to coordinate the synthesis of specific RNA and protein molecules in time and space. Morphology is a marriage of underlying laws of form and the agency of selection. 

## Conclusions

The paper deals essentially with pattern formation in biological systems trying to underline a connection between the general principles of morphogenesis, the dynamics of the reaction–diffusion systems and the fractal analysis as a tool for modeling such processes. We have considered morphogenesis as an inherently multilevel process, involving processes on different time and space scales and focusing on the reciprocal influence between these levels, showing how micro–level rules give rise (via a self–structuring process) to macro–level behavior (as in pattern formation models), but also how the macro–level behavior determines the micro–level behavior, as an essential characteristic of the living systems. Thus, in our model morphogenesis, there is no longer a slave process, but unfolds by the interactions between pattern formation, the collective behavior of the cells, and its feedback to the pattern formation process. Reaction–diffusion (RD) theory for pattern formation was considered in relation to processes of biological development in which there is a continuous growth and shape change as each new pattern forms. We have shown that RD–systems provide a strong framework for the modeling of growth processes and in particular, in biological systems. The RD–system model also permits the interaction of such systems in more complicated ways to provide emergent behaviors. Information can be considered energy, and can be manipulated as such. This may allow us a stronger method to implement bio–systems having dissipative information structures, taking into account that the same model works across multiple scales and may allow loose coupling. This is particularly common in the development of multicellular organisms that served as models for simulation, because in addition to the feedbacks in the chemical dynamics, there is then another loop linking size and shape changes with the reaction–diffusion patterning of growth controllers in the growing region. 

The research presented here can be extended in several directions. One type of possible extension to the study presented here is to better understand the behavior of the biological systems, in order to choose the most adequate mathematical model. We have already observed that much of the mathematical research that led to a large lot of models is not motivated by the study of the biological systems, and presumably will not contribute directly to their understanding. Another type of extension to the study presented here is to apply the approach of 'generic modeling' to other types of biological systems. Yet another type of possible extension is the use of the same models (or closely related) and the same bacteria in order to study various phenomena (not only biological) that are expressed in colonial pattern, and we have already started studies regarding the tumor growth [5].
